# Abnormal Type I Collagen Post-translational Modification and Crosslinking in a Cyclophilin B KO Mouse Model of Recessive Osteogenesis Imperfecta

**DOI:** 10.1371/journal.pgen.1004465

**Published:** 2014-06-26

**Authors:** Wayne A. Cabral, Irina Perdivara, MaryAnn Weis, Masahiko Terajima, Angela R. Blissett, Weizhong Chang, Joseph E. Perosky, Elena N. Makareeva, Edward L. Mertz, Sergey Leikin, Kenneth B. Tomer, Kenneth M. Kozloff, David R. Eyre, Mitsuo Yamauchi, Joan C. Marini

**Affiliations:** 1 Bone and Extracellular Matrix Branch, NICHD, NIH, Bethesda, Maryland, United States of America; 2 Laboratory of Structural Biology, NIEHS, NIH, Research Triangle Park, North Carolina, United States of America; 3 Orthopaedic Research Laboratories, University of Washington, Seattle, Washington, United States of America; 4 North Carolina Oral Health Institute, University of North Carolina, Chapel Hill, Chapel Hill, North Carolina, United States of America; 5 Orthopaedic Research Laboratories, Department of Orthopaedic Surgery, University of Michigan, Ann Arbor, Michigan, United States of America; 6 Section on Physical Biochemistry, NICHD, NIH, Bethesda, Maryland, United States of America; University of California Los Angeles, United States of America

## Abstract

Cyclophilin B (CyPB), encoded by *PPIB*, is an ER-resident peptidyl-prolyl *cis-trans* isomerase (PPIase) that functions independently and as a component of the collagen prolyl 3-hydroxylation complex. CyPB is proposed to be the major PPIase catalyzing the rate-limiting step in collagen folding. Mutations in *PPIB* cause recessively inherited osteogenesis imperfecta type IX, a moderately severe to lethal bone dysplasia. To investigate the role of CyPB in collagen folding and post-translational modifications, we generated *Ppib^−/−^* mice that recapitulate the OI phenotype. Knock-out (KO) mice are small, with reduced femoral areal bone mineral density (aBMD), bone volume per total volume (BV/TV) and mechanical properties, as well as increased femoral brittleness. *Ppib* transcripts are absent in skin, fibroblasts, femora and calvarial osteoblasts, and CyPB is absent from KO osteoblasts and fibroblasts on western blots. Only residual (2–11%) collagen prolyl 3-hydroxylation is detectable in KO cells and tissues. Collagen folds more slowly in the absence of CyPB, supporting its rate-limiting role in folding. However, treatment of KO cells with cyclosporine A causes further delay in folding, indicating the potential existence of another collagen PPIase. We confirmed and extended the reported role of CyPB in supporting collagen lysyl hydroxylase (LH1) activity. *Ppib^−/−^* fibroblast and osteoblast collagen has normal total lysyl hydroxylation, while increased collagen diglycosylation is observed. Liquid chromatography/mass spectrometry (LC/MS) analysis of bone and osteoblast type I collagen revealed site-specific alterations of helical lysine hydroxylation, in particular, significantly reduced hydroxylation of helical crosslinking residue K87. Consequently, underhydroxylated forms of di- and trivalent crosslinks are strikingly increased in KO bone, leading to increased total crosslinks and decreased helical hydroxylysine- to lysine-derived crosslink ratios. The altered crosslink pattern was associated with decreased collagen deposition into matrix in culture, altered fibril structure in tissue, and reduced bone strength. These studies demonstrate novel consequences of the indirect regulatory effect of CyPB on collagen hydroxylation, impacting collagen glycosylation, crosslinking and fibrillogenesis, which contribute to maintaining bone mechanical properties.

## Author Summary

Osteogenesis imperfecta (OI), or brittle bone disease, is characterized by susceptibility to fractures from minimal trauma and growth deficiency. Deficiency of components of the collagen prolyl 3-hydroxylation complex, CRTAP, P3H1 and CyPB, cause recessive types VII, VIII and IX OI, respectively. We have previously shown that mutual protection within the endoplasmic reticulum accounts for the overlapping severe phenotype of patients with CRTAP and P3H1 mutations. However, the bone dysplasia in patients with CyPB deficiency is distinct in terms of phenotype and type I collagen biochemistry. Using a knock-out mouse model of type IX OI, we have demonstrated that CyPB is the major, although not unique, peptidyl prolyl *cis-trans* isomerase that catalyzes the rate-limiting step in collagen folding. CyPB is also required for activity of the collagen prolyl 3-hydroxylation complex; collagen α1(I) P986 modification is lost in the absence of CyPB. Unexpectedly, CyPB was found to also influence collagen helical lysyl hydroxylation in a tissue-, cell- and residue-specific manner. Thus CyPB facilitates collagen folding directly, but also indirectly regulates collagen hydroxylation, glycosylation, crosslinking and fibrillogenesis through its interactions with other collagen modifying enzymes in the endoplasmic reticulum.

## Introduction

Type I collagen, the most abundant protein component of the extracellular matrix of skin, tendon and bone, is a heterotrimer consisting of two α1(I) and one α2(I) chains encoded by the *COL1A1* and *COL1A2* genes, respectively. The pro-alpha chains of type I collagen contain an uninterrupted helical region consisting of 338 repeats of the Gly-Xaa-Yaa triplet. Biosynthesis of procollagen is a complex process that requires several co- and post-translational modifications within the endoplasmic reticulum, including formation of disulfide bonds within the propeptide extensions, isomerization of peptidyl-prolyl bonds, hydroxylation of Yaa lysyl and prolyl residues, and glycosylation of hydroxylysines [1]. The post-translational modifications occur before, and to a major extent stabilize, collagen helical folding. After secretion into the pericellular space and processing of propeptide extensions, the mature collagen heterotrimer is incorporated into heterotypic collagen fibrils. The fibrils are then stabilized by intermolecular aldehyde-derived crosslinks formed from specific collagen lysyl and hydroxylysyl residues by lysyl oxidases (LOX) [2], [3].

Dominant mutations in *COL1A1* or *COL1A2* cause classical osteogenesis imperfecta (OI), with susceptibility to fractures from minimal trauma and growth deficiency [4]. Glycine substitutions in the collagen alpha chains delay folding and increase exposure to modifying enzymes, resulting in collagen overmodification. Some OI cases (≈10%) have recessive inheritance, caused by deficiency of proteins that interact with collagen for folding or post-translational modification [5]. Most commonly, recessive OI involves the collagen prolyl 3-hydroxylation complex, consisting of prolyl 3-hydroxylase 1 (P3H1, encoded by *LEPRE1*, leucine- and proline-enriched proteoglycan 1), cartilage-associated protein (CRTAP) and cyclophilin B (CyPB, encoded by *PPIB*, peptidyl-prolyl *cis-trans* isomerase B). This complex is responsible for 3-hydroxylation of the Xaa position α1(I) P986 residue in types I and II collagen [6]–[8]. Loss of individual components abrogates collagen P986 3-hydroxylation [8]–[11]. CRTAP and P3H1 are mutually supportive in the complex; deficiency of either component causes severe to lethal OI with rhizomelia, classified as OI types VII and VIII, respectively [12]. However, loss of the CRTAP/P3H1 complex does not decrease the level of CyPB, and, conversely, the CRTAP/P3H1 complex is only partially decreased in the absence of CyPB.

CyPB is an ER-localized member of the immunophilin family of proteins with peptidyl-prolyl *cis-trans* isomerase (PPIase) activity [13], [14]. CyPB plays a key role as a member of several foldase and chaperone complexes, including BiP, GRP94, PDI and calreticulin, for example, to facilitate folding of multiple substrates within the ER [15], [16]. *Cis-trans* isomerization of peptidyl-prolyl bonds is especially important for type I collagen folding because prolines constitute approximately one-fifth of its primary sequence. Studies published over twenty years ago demonstrated that exposure of cells to the cyclophilin inhibitor cyclosporin A (CsA) slows the rate of collagen folding and results in overmodification of lysyl residues [17]. Thus, CyPB is thought to be the major, and possibly unique, collagen peptidyl-prolyl *cis-trans* isomerase [18]. However, collagen biochemical data from the few reported patients with CyPB deficiency is inconsistent. Two patients with *PPIB* mutations causing moderate OI have normal levels of collagen prolyl 3-hydroxylation [19], [20], while in two lethal OI cases of CyPB deficiency, α1(I) P986 3-hydroxylation decreases to 30% of normal [11], [20]. Overall collagen overmodification was detected in both lethal cases, but in only one moderately severe patient.

In an attempt to clarify the inconsistencies among the human cases of PPIB/CyPB deficiency, we generated a *Ppib* knockout mouse model. We demonstrate a major, but not unique, role for CyPB in collagen folding and extend the previously reported role of CyPB in lysyl hydroxylation [21]. Biochemical studies reveal novel cell- and tissue-specific dysregulation of collagen helical lysyl hydroxylation and glycosylation in the absence of CyPB, independent of impaired collagen folding. Furthermore, reduced hydroxylation of specific collagen helical lysine residues led to a shift in the pattern of intermolecular crosslinks in bone tissue, and reduced collagen deposition into matrix. These studies establish novel functions for CyPB in regulating collagen biosynthesis and post-translational modification.

## Results

### Generation and phenotype of *Ppib*-null mice

*Ppib*-null mice were produced from an ES cell line carrying a gene trap insertion in intron 1 of *Ppib*. Two ESC lines, RST059 and RST139, were screened by real-time RT-PCR. Expression of *Ppib* in RST059 and RST139 was decreased to 76±5% and 54±3%, respectively, of wild-type levels (Figure S1A). Because RST139 ES cells were more likely to have one null *Ppib* allele, we proceeded with generation of a mouse line in the C57BL/6 background using these cells.

**Figure 1:**
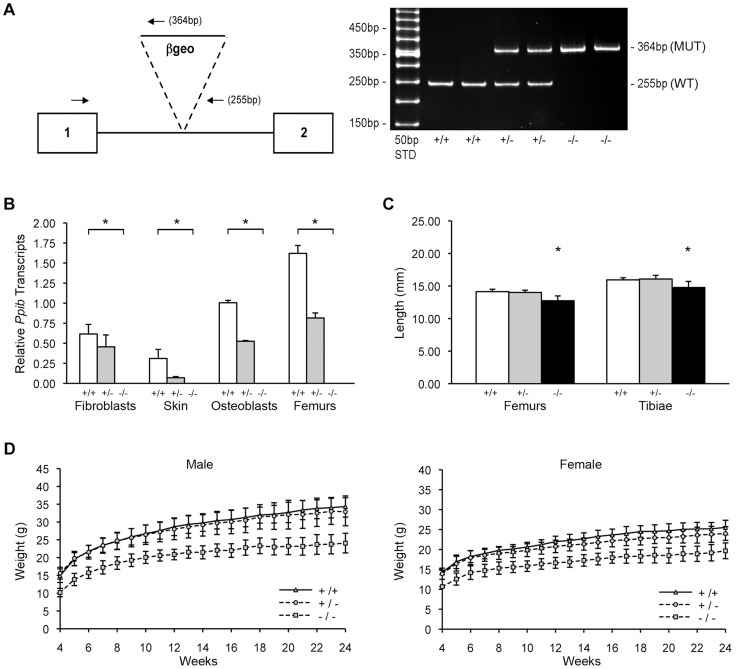
Generation of a murine model of Cyclophilin B deficiency. (A) *Left*, Diagram of knock-out allele containing β-geo reporter construct inserted into intron 1 of *Ppib* gene. Hemi-nested primers (arrows) for PCR-based genotyping selectively amplify the wild-type allele (255 bp) or the gene-trapped allele (364 bp). *Right*, Genotypes in offspring of matings between mice heterozygous for the gene-trapped allele are noted as wild-type (+/+), heterozygous (+/−) or homozygous (−/−). (B) Verification of *Ppib* expression by Real-time RT-PCR using total RNA isolated from primary cultures and tissues from newborns and 8-week mice, respectively. Values represent the average of two independent fibroblast and osteoblast cultures, and five independent dermal and femoral samples. (C) Lengths of femora and tibiae from 8 week-old wild-type (+/+), heterozygous (+/−) and homozygous (−/−) knock-out mice. Rhizomelia was not detected. (D) Growth curves of male and female mice from weaning (4 weeks) to 24 weeks of age.

Genomic DNA from homozygous mutant mice was amplified by heminested PCR (Figure 1A) and sequenced to demonstrate insertion of the gene trap vector 123 bp from the 5′-end of the1066 bp *Ppib* intron 1. The insertional mutation interrupts transcription of *Ppib* in cells and tissues isolated from *Ppib^−/−^* mice. Expression of *Ppib* in primary fibroblast (FB) and osteoblast (OB) cultures from newborn mice, as well as in dermal and femoral tissues of 8 week-old mice, was reduced in cells and tissues heterozygous for the gene-trapped allele, and completely absent from homozygous cells and tissues (Figure 1B). Thus, *Ppib* expression is completely blocked in the gene trap mutant allele.

Heterozygous mice were bred into the C57BL/6 line. In surviving homozygous offspring of F5 matings, growth deficiency became apparent soon after weaning. Knockout mice weighed about 25% less than wild-type and heterozygous littermates from 3 to 24 weeks of age (Figure 1D). At 8 weeks of age, *Ppib^−/−^* femoral and tibial lengths were reduced 7% and 10% versus wild-type mice and heterozygotes (p<0.00004 and p<0.02, respectively) (Figure 1C). However, in contrast to *Crtap* and *P3H1* null mice [8], [22], *Ppib^−/−^* mice do not have rhizomelia. The ratio of femoral to tibial length is comparable to wild-type (0.886±0.020 vs 0.861±0.035, p = 0.07).

### *Ppib* is required for bone development

**Figure 2:**
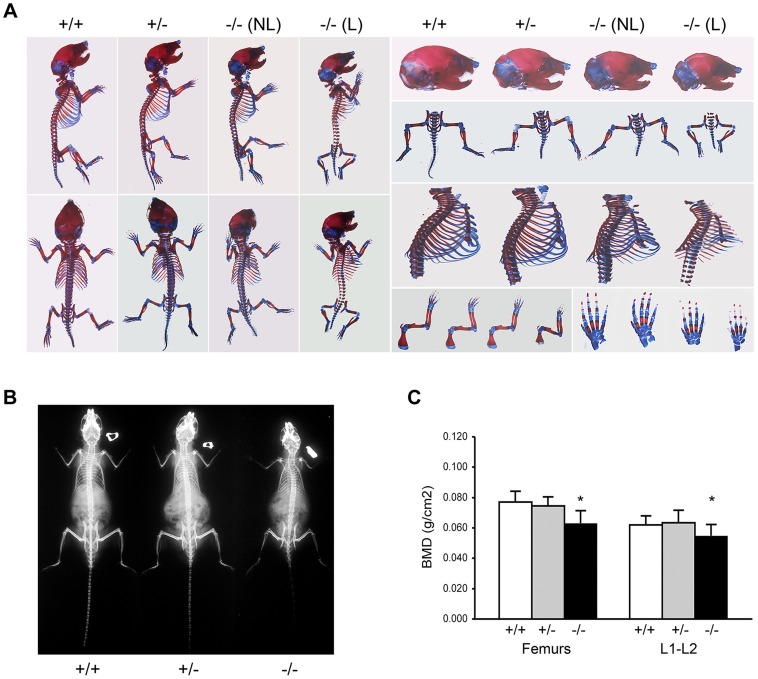
Absence of *Ppib* expression affects bone development. (A) Staining of newborn skeletons with Alizarin red (bone) and Alcian blue (cartilage) reveals undermineralization of calvaria and ribs. Homozygous mice have smaller size of whole skeleton and long bones, and a deformed rib cage. (B) X-rays of 8 week-old mice. (C) DXA analysis of 8 week-old mice (n = 10/genotype).

Skeletal abnormalities of *Ppib^−/−^* mice include decreased mineralization and abnormal shape of calvaria, shortened limbs and a deformed and flared rib cage (Figure 2A); these features are accentuated in lethal null pups. The genotype distribution among offspring of F4 and F5 het x het crosses deviated from the Mendelian ratio, with 30 and 50% lethality of homozygous pups from F4 and F5 matings, respectively, likely due to respiratory insufficiency from abnormal rib cage structure (Figure 2A). Heterozygous mice are essentially normal. At 2 months of age, the rib cage of both heterozygous and homozygous mice has a narrow apex and drooping ribs at the base, providing limited space under the ribs for abdominal contents, which puff out the abdomen (Figure 2B). Homozygous mice also have kyphosis.

**Figure 3:**
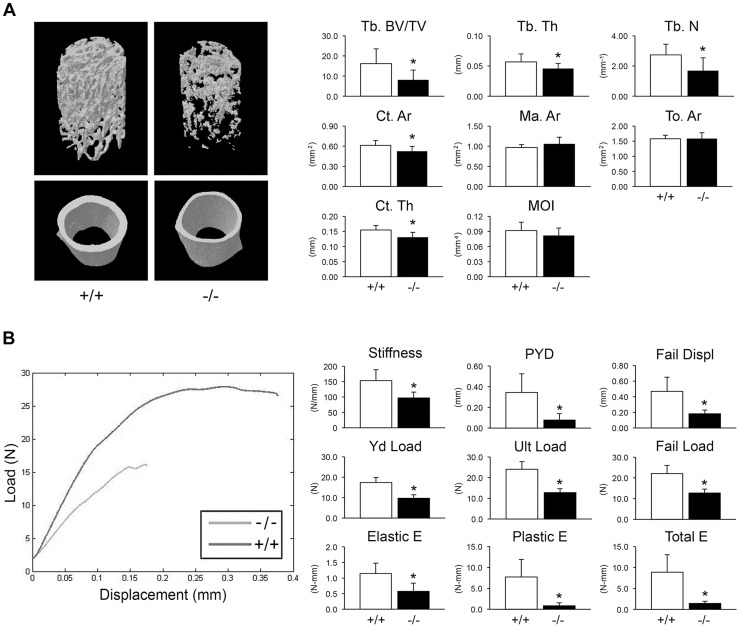
Whole bone structural and mechanical properties. (A) Structural parameters of wild-type (+/+) and homozygous (−/−) femora at 8 weeks of age characterize reduced bone formation in CyPB-deficient mice (n = 9/genotype). *Left*, 3D reconstructions illustrate reduced trabecular and cortical bone volumes. *Right*, Trabecular parameters are decreased in homozygous (−/−) femora, including reduced bone volume (Tb BV/TV, p = 0.01), thickness (Tb Th, p = 0.04) and number (Tb N, p = 0.01). Cortical bone parameters of CyPB-deficient mice are also reduced, with reductions in cortical thickness (Ct Th, p = 0.003) and area (Ct Ar, p = 0.02). (B) *Left*, Representative load-displacement curve demonstrating differences between samples selected for median post-yield displacement. *Right*, CyPB-deficient femora are weaker in yield (Yd Load, p = 1.9×10^−6^), ultimate (Ult Load, p = 8.9×10^−7^) and failure loads (p = 2.1×10^−5^), with reduced stiffness (p = 0.001). *Ppib* ^−/−^ femora are also more brittle than wild-type femora, as demonstrated by decreased post-yield displacement (PYD, p = 0.001). Reduced toughness of *Ppib* ^−/−^ femora is evident by decreases in the elastic and plastic energy (E) values (p = 0.001 and 0.0003, respectively).

Adult knockout mice are osteoporotic; they have decreased aBMD of femora (p = 0.001) and vertebrae (p = 0.02) (Figure 2C). Femora of 2-month male *Ppib^−/−^* mice display altered cortical and trabecular structure on μCT analysis (Figure 3A; Table S1). Their trabecular bone volume is half of wild-type, with reduction of both trabecular number and thickness. Femoral cortical bone is thinner, with decreased cortical area and a modestly enlarged marrow space.

Femora were mechanically tested to assess fragility. *Ppib* ^−/−^ femora have reduced stiffness, yield load, and ultimate load, requiring 48% less total energy to fracture than wild-type controls (Figure 3B). Femoral post-yield displacement and plastic energy were reduced 77% and 89% respectively, demonstrating a more brittle phenotype characteristic of OI. Importantly, while stiffness was reduced 37% in *Ppib* ^−/−^ mice (p<0.01), cortical bending moment of inertia, as measured by microCT, was only modestly reduced (−11%, p = 0.14) suggesting there may be significant changes in bone material properties at levels unaccounted for by changes in cortical geometry.

### CyPB contributes to prolyl 3-hydroxylation complex structure and function

**Figure 4:**
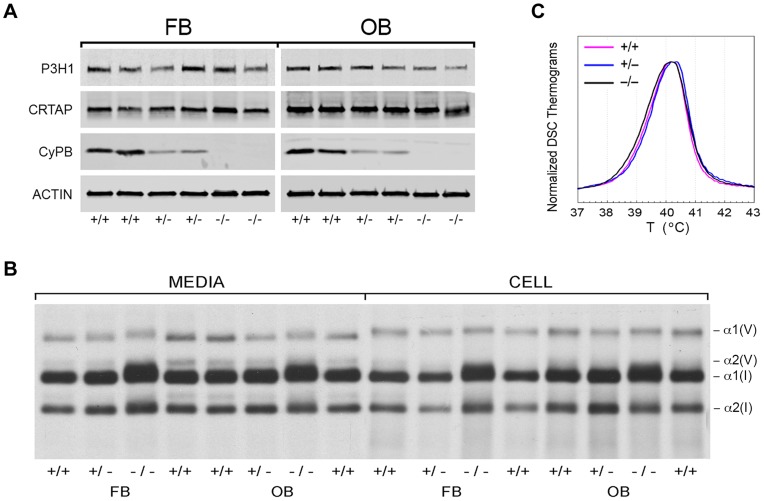
Synthesis of type I collagen. (A) Western blots of cell lysates with antibodies to collagen 3-hydroxylation complex components. Lysates are derived from two independent cultures for each genotype. (B) SDS-Urea PAGE analysis of steady-state labeled type I collagen from wild-type (+/+), heterozygous (+/−) and homozygous (−/−) fibroblasts (FB) and osteoblasts (OB). (C) Differential scanning calorimetry (DSC) analysis reveals no differences in thermal stability (T_m_) of type I collagen secreted by fibroblast cultures.

We examined the components of the collagen 3-hydroxylation complex in CyPB-deficient cells. In *Ppib* ^−/−^ OB but not FB, complete absence of CyPB was associated with a 40–55% reduction in P3H1 (Figure 4A). CRTAP levels were not altered in either cell type. Although CyPB was decreased in heterozygous cells, P3H1 and CRTAP were unchanged. Despite the persistence of P3H1 and CRTAP in *Ppib^−/−^* cells, 3-hydroxylation of α1(I) P986 residues was severely reduced. In *Ppib^−/−^* mice, only 5–11% of OB and FB and 1–2% of dermal and bone tissue collagen α1(I) P986 residues were modified, in contrast to wild type and heterozygous cultures and tissues in which there is nearly complete collagen 3-hydroxylation (Table 1). The secondary prolyl 3-hydroxylation site was also undermodified; *Ppib*-null OB and femora have half the α2(I) P707 3-hydroxylation of wild-type and heterozygous samples.

**Table 1:** Type I collagen post-translational hydroxylation.

	Hyl/(Hyl+Lys)	4-Hyp/(Hyp+Pro)	% α2(I) P707 3-Hyp	% α1(I) P986 3-Hyp	% Telo Hyl (NH2, COOH)
**+/+**					
fibroblast	20.0±1.5	49.5±1.3	<10	97	ND
skin	12.9±1.2	40.2±2.2	6–10	80	ND
osteoblast	46.4±1.8	50.9±1.5	24	97	ND
femur	ND	ND	13–21	98	85, 85
humerus	24.8±3.2	45.4±1.2	ND	ND	ND
**+/−**					
fibroblast	21.7±0.8	48.5±0.9	<10	>89	ND
skin	12.8±0.3	39.8±0.8	6–11	80	ND
osteoblast	ND	ND	24	97	ND
femur	ND	ND	10–12	98	ND
humerus	30.1±0.2	46.1±1.4	ND	ND	ND
**−/−**					
fibroblast	20.1±1.0	51.2±1.2	<10	11	ND
skin	2.5±1.9	41.9±1.4	1–2	2	ND
osteoblast	43.7±0.8	52.7±1.5	13	5	ND
femur	ND	ND	5–7	2	95, 83
humerus	30.8±3.4	45.1±1.1	ND	ND	ND

ND, Not determined.

### CyPB role in collagen folding and helical modification

Type I collagen alpha chains from *Ppib^−/−^* FB and OB have delayed and broadened gel migration, consistent with delayed folding and overmodification of lysyl residues (Figure 4B). Collagen overmodification in the absence of a structural defect is expected to increase thermal stability [10], [23]. However, type I collagen secreted by wild-type, heterozygous and homozygous null cells had equivalent melting temperatures (T_m_) by differential scanning calorimetry (Figure 4C). In agreement with T_m_ results, amino acid analysis of type I collagen secreted by *Ppib^−/−^* FB and OB revealed normal proportions of hydroxylated lysine and proline residues (Table 1).

**Figure 5:**
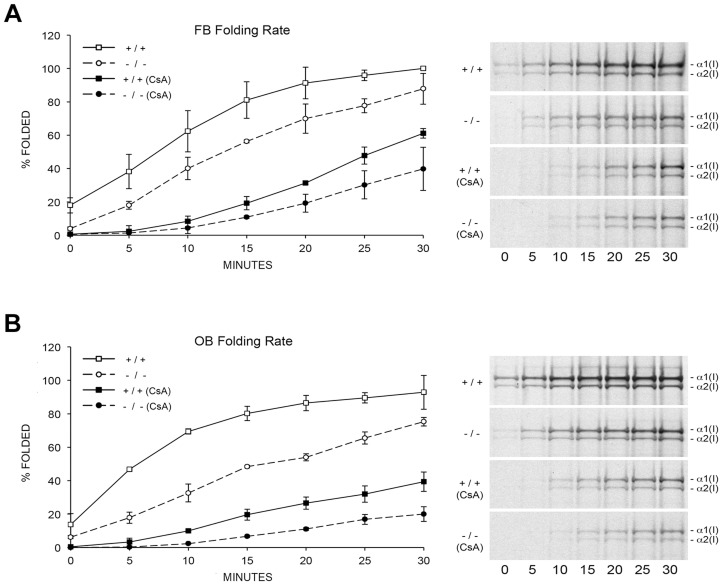
Cyclophilin B catalyzes folding of type I collagen. (A) Assay for intracellular folding of type I collagen in fibroblast cultures. (B) Assay for intracellular folding of type I collagen in calvarial osteoblast cultures. Data represents the average from two independent cell lines for each genotype.

To resolve these apparent discrepancies, we utilized a direct intracellular collagen folding assay. Accumulation of intracellular protease-resistant collagen was slower in *Ppib-*null FB than in wild-type cells (Figure 5A), with a greater percent of folded trimers in wild-type cells throughout the independent time course experiments. In *Ppib^−/−^* OB, the delay in type I collagen folding compared to wild-type was nearly double that in FB (Figure 5B). Notably, addition of the inhibitor CsA to both fibroblasts and osteoblasts further delayed the rate of collagen folding in *Ppib^−/−^* as well as wild-type cells. These data suggest that, although CyPB is the primary PPIase involved in catalyzing folding of the type I collagen helix, other PPIases may also be capable of supporting this function. Furthermore, since collagen folds more slowly in *Ppib* ^−/−^ than wild-type cells both with and without CsA treatment, two PPIases and/or a CyPB-dependent protein may be involved in collagen folding.

### CyPB role in site-specific regulation of collagen post-translational modifications

**Figure 6:**
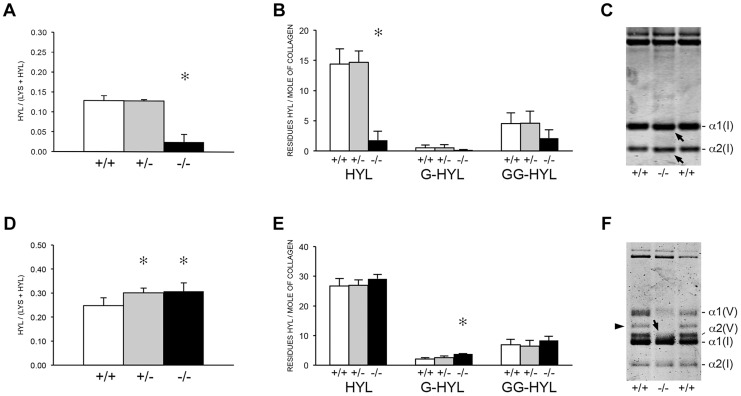
Post-translational modification of type I collagen from tissues. (A) Total lysyl hydroxylation of type I collagen is dramatically reduced in dermal tissue from *Ppib* ^−/−^ mice (p<0.001 vs wild-type). (B) Post-translational hydroxylation and glycosylation of type I collagen lysyl residues in dermal tissue from *Ppib* ^−/−^ mice. (C) SDS-Urea PAGE analysis of pepsin extracts from dermal tissue demonstrates increased electrophoretic migration of *Ppib* ^−/−^ type I collagen alpha chains compared to wild-type. Arrows indicate faster migrating alpha chains. (D) Total lysyl hydroxylation of type I collagen extracted from bone tissue of heterozygous (+/−) and homozygous (−/−) *Ppib*-null mice compared to wild-type (+/+) bone collagen. Both *Ppib* ^+/−^ and *Ppib* ^−/−^ bone collagen show increased Hyl compared to wild-type (p = 0.0002 and 0.005, respectively). (E) Analysis of post-translational lysine hydroxylation and glycosylation in *Ppib* ^−/−^ bone-derived type I collagen demonstrates increased galactosyl-hydroxylysine (G-HYL) content compared to wild-type (p<0.001). (F) Type I collagen extracted from bone tissue displays backstreaking of α1(I) chains on SDS-Urea, indicated by arrow, and is consistent with post-translational overmodification. Arrowhead indicates a truncated form of α1(V) chains due to pepsin sensitivity.

The apparent discrepancy between slower folding rate and normal total lysyl hydroxylation of collagen from *Ppib^−/−^* mice led to a more detailed analysis of collagen hydroxylation and subsequent glycosylation in null cells and tissues. Biochemical analysis of *Ppib^−/−^* skin tissue showed a substantial decrease in lysyl hydroxylation (18% of wild-type) (Figure 6A) and glycosylation (40–50% of wild-type) (Figure 6B) in collagen. A site-specific analysis of skin and fibroblast lysine modification and crosslink patterns will be presented elsewhere. Decreased modification of skin-derived type I collagen resulted in slightly faster gel migration (Figure 6C). However, *Ppib* ^−/−^ bone collagen lysyl hydroxylation was slightly increased in heterozygous (p = 0.0002) and homozygous (p = 0.005) mice (Figure 6D), with a significant increase in galactosylhydroxylysine (p<0.001) in homozygous mice (Figure 6E) and subtly broadened electrophoretic mobility (Figure 6F). In addition, *Ppib* ^−/−^ bone extracts show a substantial decrease in type V collagen alpha chains.

**Figure 7:**
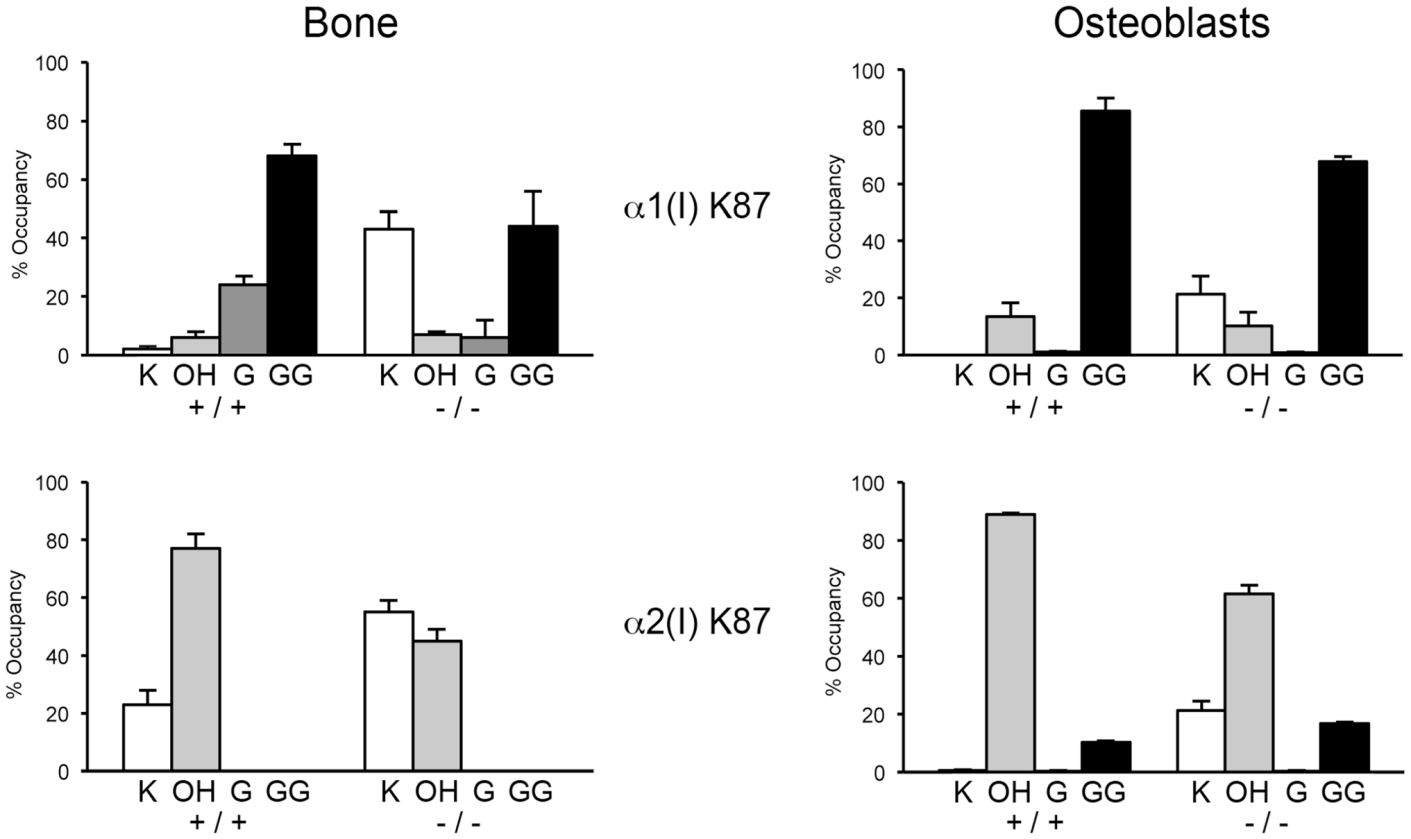
Altered post-translational modification of specific type I collagen lysine residues in the absence of Ppib. Quantitation of type I collagen modifications at α1(I) and α2(I) K87 residues, which are important for crosslinking of type I collagen heterotypic fibrils in tissue. Results were obtained from collagen secreted in culture by primary osteoblasts, or from 3–5 independent tissue samples for each genotype. K, lysine; OH, hydroxylysine; G, galactosylhydroxylysine; GG, glucosylgalactosylhydroxylysine.

Detailed characterization of lysine residues in collagen from OB cultures and bone tissue showed site-specific changes in lysine hydroxylation and glycosylation, and differences between cultured cells and tissues (Table 2; Figure 7). In *Ppib^−/−^* OB collagen, only α1(I) K87, α2(I) K87 and K174 are underhydroxylated, with about 20% of K87 residues in *Ppib^−/−^* collagen unhydroxylated versus <1% of wild-type. However, hydroxylation of other helical lysines from *Ppib^−/−^* OB collagen was normal or subtly increased, i.e. α1(I) K603 and K756 hydroxylation were increased 14% and 9%, respectively (Table 2). Bone tissue collagen from *Ppib^−/−^* mice has an even more striking increase in the proportion of lysine residues involved in crosslink formation that are unhydroxylated. Hydroxylation of α2(I) and α1(I) K87 residues is decreased 30–40%, respectively, and α2(I) K933 hydroxylysine content is decreased 38%, compared to wild-type bone (Table 2). Decreased hydroxylation is also evident at α2(I) K174, but other residues do not have significant changes.

**Table 2:** Post-translational modification of type I collagen lysine residues.

		BONE			OB	
		+/+	+/−	−/−	+/+	−/−
α1(I) K87	Lys	2.0±1.0	2.0±0.4	43.0±6.0* #	0.1±0.1	21.2±6.4*
	Hyl	6.0±2.0	13.0±5.0	7.0±1.0#	13.4±4.8	10.1±4.7
	G-Hyl	24.0±3.0	22.0±7.0	6.0±6.0* #	0.9±0.3	0.8±0.1
	GG-Hyl	68.0±4.0	63.0±11.0	44.0±12.0* #	85.6±4.6	67.7±1.8*
α1(I) K174	Lys	18.0±1.0	19.0±3.0	15.0±2.0	15.5±2.7	9.3±1.9*
	Hyl	67.0±2.0	66.0±2.0	56.0±1.0* #	54.2±4.2	33.4±1.4*
	G-Hyl	12.0±2.0	13.0±1.0	19.0±1.0* #	9.7±3.4	8.9±2.9
	GG-Hyl	2.0±0.0	2.0±0.0	10.0±2.0* #	20.5±3.5	48.3±2.9*
α1(I) K564	Lys	0.0	0.3±0.2	0.0	11.0±5.6	9.8±2.4
	Hyl	76.0±4.0	79.0±3.0	45.0±5.0* #	56.3±1.3	44.9±4.9*
	G-Hyl	21.0±4.0	17.0±2.0	31.0±3.0#	13.7±2.5	13.4±2.6
	GG-Hyl	3.0±1.0	4.0±1.0	24.0±4.0* #	18.9±3.0	31.9±4.7*
α1(I) K603	Lys	42.0±3.0	40.0±1.0	37.0±2.0	42.0±4.6	28.2±4.3*
	Hyl	57.0±3.0	59.0±1.0	58.0±2.0	49.1±5.4	48.0±4.8
	G-Hyl	0.6±0.1	0.6±0.0	2.0±0.0*	1.9±0.1	4.1±0.6*
	GG-Hyl	0.2±0.1	0.4±0.0	2.0±1.0* #	7.0±1.4	19.6±2.1*
α1(I) K756	Lys	40.0±2.0	41.0±1.0	41.0±5.0	40.5±6.8	31.5±3.4
	Hyl	59.5±3.0	58.5±0.8	57.0±6.0	54.5±6.9	57.1±1.9
	G-Hyl	0.4±1.0	0.5±0.2	1.5±1.0	1.2±0.1	2.3±0.3*
	GG-Hyl	0.1±0.1	0.1±0.1	0.6±0.5	3.7±0.2	10.8±0.9*
α2(I) K87	Lys	23.0±5.0	20.0±4.0	55.0±4.0* #	0.6±0.1	21.3±3.1*
	Hyl	77.0±5.0	80.0±4.0	45.0±4.0* #	88.9±0.7	61.6±3.0*
	G-Hyl	0.0	0.0	0.0	0.3±0.1	0.3±0.1
	GG-Hyl	0.0	0.0	0.0	10.2±0.6	16.8±0.4*
α2(I) K174	Lys	25.0±3.0	20.0±8.0	38.0±6.0* #	24.1±3.0	42.0±2.4*
	Hyl	19.0±3.0	20.0±2.0	8.0±3.0* #	15.3±2.1	4.1±0.6*
	G-Hyl	51.0±3.0	53.0±6.0	38.0±4.0* #	23.4±2.3	8.9±0.6*
	GG-Hyl	5.0±3.0	7.0±2.0	16.0±2.0* #	37.3±2.3	45.0±2.1*
α2(I) K219	Lys	9.0±2.0	8.0±2.0	8.0±2.0	13.3±0.6	7.8±0.6*
	Hyl	86.0±1.0	86.0±2.0	75.0±3.0* #	73.6±5.8	50.9±1.0*
	G-Hyl	4.0±1.0	4.0±1.0	5.0±1.0	0.8±0.3	1.2±0.3
	GG-Hyl	1.0±0.6	2.0±0.0	12.0±4.0* #	12.3±5.9	40.1±1.6*
α2(I) K933	Lys	0.0	0.0	38.0±3.0* #	2.9±0.2	0.0*
	Hyl	100.0	100.0	62.0±3.0* #	92.0±2.8	91.2±1.9
	G-Hyl	0.0	0.0	0.0	1.0±0.4	0.9±0.2
	GG-Hyl	0.0	0.0	0.0	4.0±2.4	7.9±1.7

*p<0.05 between +/+ and −/−;

# p<0.05 between +/− and −/−;

no significant difference between +/+ and +/− in bone.

Glycosylation patterns are also altered in *Ppib^−/−^* collagen. In wild-type OB collagen, a major helical cross-linking residue, α1(I) K87, has the highest proportion of diglycosylation, while α2(I) K87 is mainly non-glycosylated (Table 2 and Figure 7), as we previously reported [24], [25]. In *Ppib^−/−^* collagen, mono- and di-glycosylation of α1(I) K87 is decreased in tissue and OB culture collagen, and α2(I) K87 di-glycosylation is only slightly increased in OB culture collagen, despite slow helical folding (Figure 7). In OB and bone-derived *Ppib^−/−^* collagen, all other lysine residues assayed have substantial increases in glycosylation, corresponding with delayed intracellular collagen folding. We observed increased glycosylation at α1(I) K174, K564, K603, K756 and α2(I) K219 in *Ppib^−/−^* OB collagen, and to a lesser extent in *Ppib^−/−^* bone-derived collagen, (Table 2), in agreement with the increased total lysyl hydroxylation and broad gel migration (Figure 6D–F).

**Figure 8:**
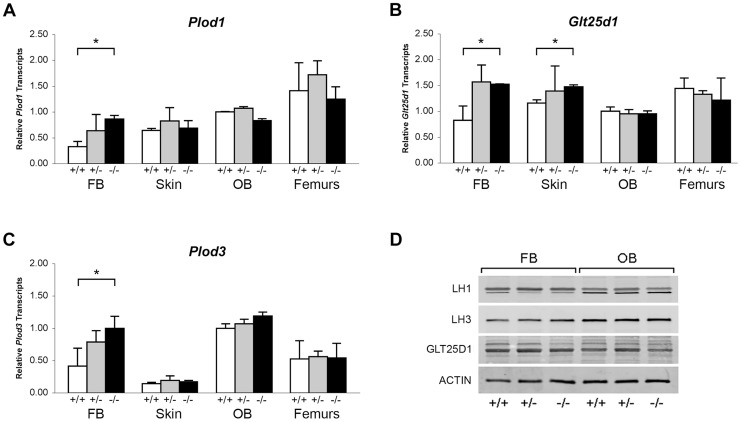
Expression of ER resident collagen helical lysine modification enzymes. (A–C) Real-time RT-PCR of total RNA from two independent cell cultures of newborn fibroblasts (FB) and calvarial osteoblasts (OB), and 5 independent skin and femoral samples for each genotype at 8 weeks of age. (D) Western blots of cell lysates probing for lysyl hydroxylase 1 (LH1/PLOD1), lysyl hydroxylase 3 (LH3/PLOD3), glycosyltransferase 25 domain containing 1 (GLT25D1), and β-actin in wild-type (+/+), heterozygous (+/−) and homozygous (−/−) *Ppib*-null cells and tissues.

Collagen helical lysine modifications are catalyzed primarily by lysyl hydroxylase 1 (LH1) for hydroxylation, GLT25D1, for galactosylation of hydroxylysine [26], and LH3, which harbors both lysyl hydroxylase and glucosyltransferase activities [24], [27]–[29]. By real-time RT-PCR, we found a modest increase in transcript levels for all three enzymes in *Ppib^−/−^* FB cultures, but not in skin, OB or femoral tissue (Figure 8A–C). Importantly, the protein levels of these enzymes were normal on western blots of FB and OB lysates (Figure 8D). These data verify that expression levels of the modifying enzymes do not account for the alterations in lysine modification demonstrated in collagen from *Ppib* ^−/−^ cells and tissues.

### Absence of CyPB affects collagen synthesis and secretion

**Figure 9:**
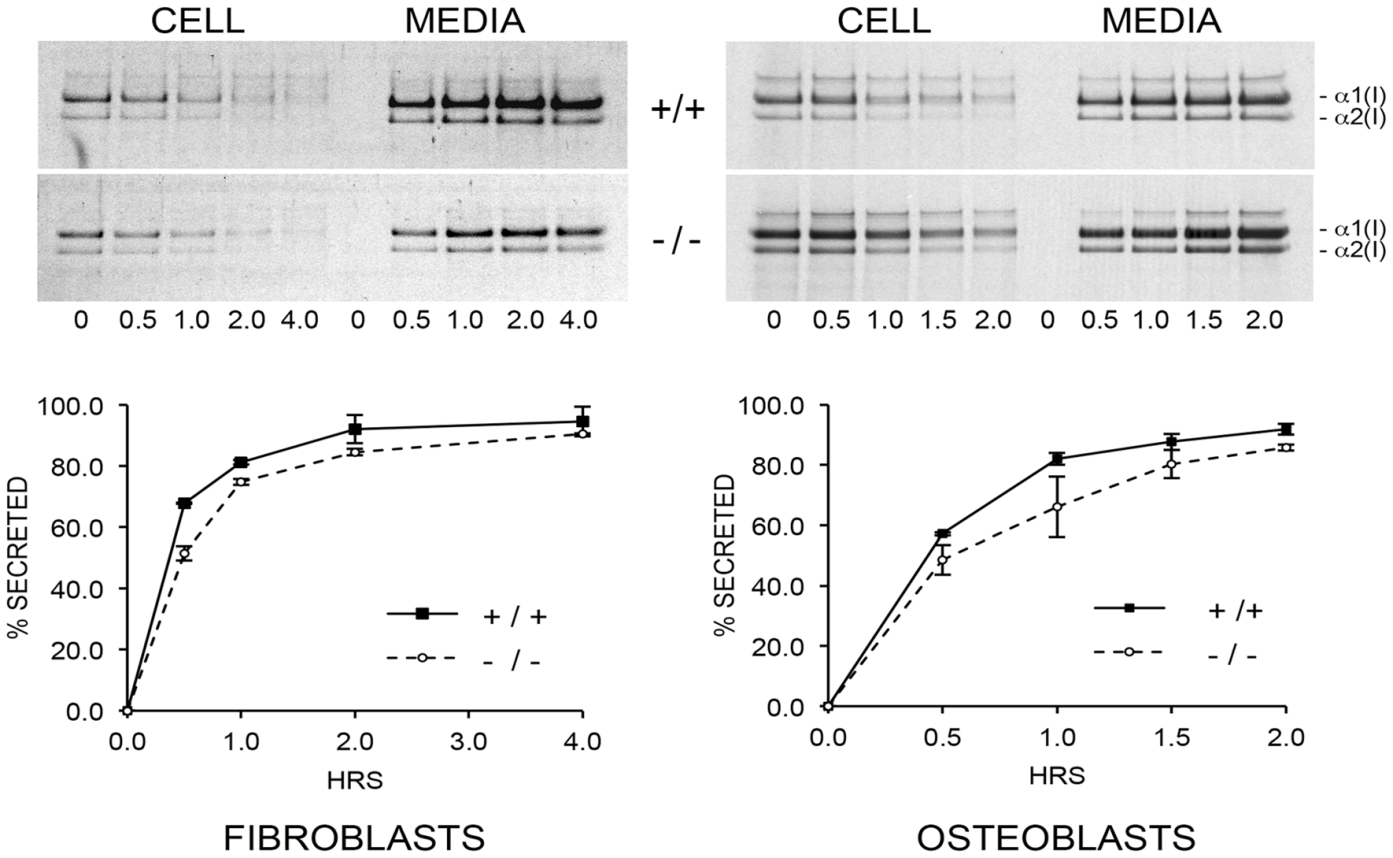
Pulse-chase analysis of type I collagen secretion. There is a minimal delay in secretion of collagen by cyclophilin B-deficient fibroblasts and osteoblasts in culture.

The combined effects of CyPB absence, slower collagen folding and abnormal modification, impact procollagen synthesis. Pulse-chase experiments show increased (nearly double) total collagen synthesis per cell by CyPB-deficient OB, while fibroblasts produce about half the amount of collagen per cell as wild-type cells (Figure 9, upper). However, the kinetics of collagen secretion is only slightly delayed for *Ppib^−/−^* FB and OB, (Figure 9, lower), but of uncertain physiological significance, since nearly the entire pulse of labeled collagen is secreted in the same timeframe as wild-type cells. This finding is similar to our previous report on FB with null mutations in *LEPRE1* [10], but distinct from the reported increased rate of collagen secretion in FB with null mutations in *CRTAP* [8].

### Absence of CyPB affects collagen matrix deposition and crosslinking

**Figure 10:**
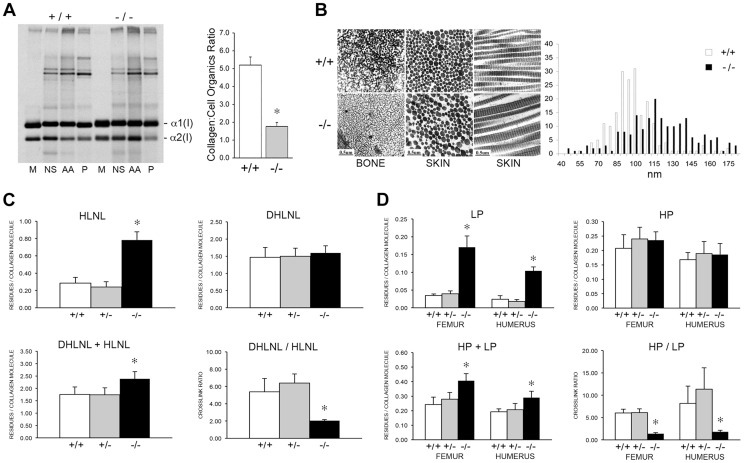
Dysregulation of collagen deposition and fibril assembly. (A) *Left*, Deposition of type I collagen by osteoblasts into extracellular matrix in culture. Post-confluent cultures were pulsed for 24 hr, followed by serial extraction of incorporated collagens from the media (M), neutral salt (NS), acid soluble (AA, immaturely crosslinked) and pepsin soluble (P, maturely crosslinked) fractions of the matrix. *Right*, Matrix collagen to cell organics ratio from Raman micro-spectroscopy shows decreased collagen content in matrix deposited by homozygous *Ppib*-null (−/−) versus wild-type (+/+) osteoblasts in culture (p = 0.002). (B) Transmission electron micrographs of femoral and dermal collagen fibrils from 8 week-old wild-type (+/+) and CyPB-deficient mice (−/−). Diameters of 200 dermal fibrils were measured for each sample and plotted, right. (C) Quantitation of divalent crosslinks in murine humeri reveals increased HLNL (hydroxylysinonorleucine) crosslinks, which require helical lysine residues, but no change in DHLNL (dihydroxylysinonorleucine) crosslinks, which involve helical hydroxylysine residues. (D) Quantitation of trivalent crosslinks in murine humeri and femora. Total pyridinoline crosslinks are increased due to an increase in lysyl pyridinoline (LP), but not hydroxylysyl pyridinoline (HP) crosslinks in bone.

To determine the consequences of altered collagen post-translational modification on extracellular matrix, we analyzed collagen deposition into matrix in culture. Sequential extraction of the incorporated labeled collagen revealed that collagen deposited into insoluble matrix by OB (Figure 10A, left) and FB (Figure S1B) was decreased 80% and 70%, respectively, compared to wild-type cultures. Raman spectroscopy of OB-derived matrix corroborated the biochemical analysis (Figure 10A, right), indicating a two-thirds reduction in matrix collagen content compared to wild-type.

Consistent with the marked reduction in hydroxylation of the helical crosslinking lysine in bone in the absence of CyPB, there was a significant difference in the crosslinking pattern. For divalent crosslinks, there was a nearly three-fold increase in the helical lysine-involved crosslink, hydroxylysinonorleucine (HLNL), in homozygous humeral bone (p = 1.9×10^−8^), while the helical hydroxylysine-involved dihydroxylysinonorleucine (DHLNL) is comparable to wild-type bone (Figure 10C). The increase in HLNL crosslinks decreases the DHLNL/HLNL ratio in *Ppib* ^−/−^ humeri (p = 0.0004). For the trivalent mature crosslinks, hydroxylysylpyridinoline (HP) crosslinks were unchanged in *Ppib^−/−^* bone relative to wild-type. However, the helical lysine-involved crosslink, lysylpyridinoline (LP), was markedly increased by four to five-fold in *Ppib^−/−^* humerii (p = 2.8×10^−9^) and femoral tissue (p = 0.0001), respectively (Figure 10D). The resulting HP/LP ratio in CyPB-deficient bone is decreased 4.25-fold in humeral (p = 0.00004) and 5.6-fold in femoral bone tissue (p = 0.003). It is also quite noteworthy, in the context of both the reduced collagen deposition into *Ppib^−/−^* osteoblast matrix and the increase in non-hydroxylated forms of crosslinks in tissue, that total bone collagen crosslinks were increased in null mice versus wild-type at 2 months of age (DHLNL+HLNL, p = 0.002; HP+LP, p = 0.00004 and 0.003 for humerus and femora, respectively).

The abnormal collagen modification and crosslinking affects the structure and organization of collagen fibrils. In dermal fibrils of *Ppib^−/−^* mice, we noted the presence of disorganized aggregate forms, as well as a 25% increase in the average fibril diameter (p = 1.7×10^−15^), and a broader distribution of fibril diameters (p = 4.0×10^−8^) (Figure 10B). In contrast to dermal fibrils, collagen fibrils from femoral tissue were less densely packed and had visibly decreased diameters compared to wild-type. Bone fibril diameters were not quantitated because multiple bundle orientations in each field does not allow a clear analysis of fibril cross-sections.

## Discussion

To investigate the role of CyPB/PPIB in post-translational modification and folding of type I collagen, we generated a CyPB knockout mouse using a *Ppib* gene-trapped ES cell line. We confirmed complete absence of *Ppib* transcripts in cultured cells, as well as dermal and bone tissue of homozygous null mice. Furthermore, CyPB was undetectable in immunoblots of both fibroblast and osteoblast cultures. The resulting murine phenotype reproduces the clinical findings in patients with *PPIB* deficiency, including growth deficiency with bone deformities, reduced bone mineral density, decreased bone volume and strength. Interestingly, the impairment of mechanical properties of *Ppib* ^−/−^ femora exceeded the extent expected from bone structural parameters, suggesting that bone material properties have also declined.

This study recapitulates some features previously reported in a CyPB-deficient mouse, including a moderately severe skeletal phenotype with reduced bone geometry, absence of α1(I) P986 3-hydroxylation and delayed collagen migration on gel electrophoresis. The prior study focused on the role of CyPB in the P3H complex, while we investigated CyPB's function in collagen synthesis, folding and helical modification. We found that collagen folding is delayed in *Ppib^−/−^* cells, and further folding delay by CsA inhibition provides support for an additional collagen PPIase. Furthermore we identified striking differences in the pattern of collagen post-translational modification in cells versus tissues. Unexpectedly, CyPB deficiency also results in tissue- and site-specific alterations of post-translational hydroxylation and glycosylation of type I collagen, which consequently affect fibril assembly, crosslinking in matrix, and bone mineralization. Thus our study offers novel mechanisms with the potential to explain the unique features of CyPB deficiency in humans, compared to the consequences of P3H1/CRTAP defects.

In our *Ppib* KO mice, α1(I) P986 3-hydroxylation is nearly absent from dermal and bone collagen, demonstrating that CyPB is required *in vivo* for 3-hydroxylation complex activity in tissues. This is consistent with the 3-hydroxylation status previously reported in murine *Ppib*-null bone and skin collagen, as well as with <1% modification of P986 residues in α1(I), α1(II) and α2(V) chains from bone, skin and cartilage of *Crtap*-null mice [8], [30], [31]. P986 3-hydroxylation of type I collagen was also absent in tail tendon, bone and skin of P3H1-null mice [22], [32]. At the A2 site (α2(I) P707), we observed a 50% reduction of 3-hydroxylation in collagen from skin and bone, suggesting that P3H1 or other P3H isoforms can modify this site without requiring CyPB for activity. For other sites and/or collagen alpha chains, the requirement for CyPB may be quite stringent. Delayed electrophoretic migration of collagen alpha chain, caused by increased post-translational modification, is considered to be a reliable surrogate assay for slower helix folding. Steady-state collagen from *Ppib^−/−^* cultured fibroblasts and osteoblasts has delayed gel mobility, consistent with the significant delay in helical folding of type I collagen demonstrated in direct intracellular folding assays in both cell types. Collagen folding in *Ppib* ^−/−^ fibroblasts and osteoblasts was delayed 5 to 8 minutes, respectively. By comparison, fibroblast cell lines from OI patients harboring typical structural defects in type I collagen demonstrated 5 to 60 minutes delay in formation of the collagen helix versus control cells, depending on the glycine substitution [33].

Treatment of control fibroblast and osteoblast cultures with cyclosporin A (CsA), a non-specific cyclophilin inhibitor, resulted in a 10–12 minute delay in collagen folding, similar to published experiments [17], [34]. Unexpectedly, CsA treatment of CyPB-deficient cells further increased the collagen folding delay by 6–8 minutes beyond that seen in untreated cells. These data suggest that another CsA-sensitive PPIase is capable of partial compensation for CyPB deficiency, and may be involved in collagen folding under normal conditions. Furthermore, even in CsA treated cells, folding remains slower in *Ppib* ^−/−^ than WT cells, suggesting that a CyPB-dependent, CsA-resistant function may also be supporting collagen folding. We previously reported a type IX OI patient with total absence of CyPB due to a start codon substitution [19]. Fibroblast collagen from this patient had normal helical modification suggestive of normal collagen folding, prompting us to propose redundancy of collagen *cis*-*trans* isomerases. The identity of this PPIase is open to speculation. Only one cyclophilin, but at least six FK506 binding proteins (FKBPs), occur in the rER [13], [14]. Until recently, FKBP65, which binds gelatin and is partially inhibited by CsA *in vitro*, was an attractive candidate [35]. However, addition of FK506 to cell cultures delays collagen folding only slightly [34], and absence of FKBP65 in patients with OI, Bruck syndrome and Kuskokwim disease has negligible effects on collagen helical folding [36]–[38], limiting FKBP65 to at most a compensatory role in the absence of CyPB. Similarly, mutations in *FKBP14*, which encodes FKBP22, were identified in an Ehlers-Danlos like-syndrome similar to the kyphoscoliotic type of EDS (EDS VIA) caused by LH1 deficiency, but their types I, III and V collagens showed normal electrophoretic migration [39].

One of the striking findings of this study was the difference in post-translational modification in collagen isolated from cell cultures versus dermis and bone. *Ppib^−/−^* fibroblast and osteoblast steady-state type I collagen has significant electrophoretic delay, but normal total hydroxylysine content, which suggested that increased glycosylation delayed gel mobility. In contrast, collagen extracted from dermal tissue of CyPB-deficient mice has an 80% decrease in total helical hydroxylysine content, leading to more rapid gel migration of alpha chains than collagen from wild-type skin. The decreased hydroxylation of collagen in dermal tissue of the *Ppib* ^−/−^ mouse is similar to the dermal tissue data from American quarter horses with hyperelastosis cutis caused by a homozygous *Ppib* missense mutation that does not impair its isomerase function *in vitro*. The significantly decreased hydroxylysine content of dermal collagen in the horse is proposed to result from loss of a CyPB-LH1 interaction required for LH1 activity [21].

Type I collagen extracted from humeri of *Ppib* ^−/−^ mice had different post-translational modification than both osteoblasts and dermal tissue. Bone-derived collagen alpha chains demonstrated subtle broadening and backstreaking on PAGE analysis, consistent with the increased global hydroxylation and glycosylation of lysyl residues determined by amino acid analysis. However, detailed mass spectrometric analysis revealed site-specific alterations in lysine post-translational modification of bone tissue collagen. We found dramatic underhydroxylation of lysine residues involved in the formation of intermolecular crosslinks in tissue, specifically at α1(I) K87 (57% Hyl in null vs 98% in WT), α2(I) K87 (45% Hyl in null vs 77% in WT), and α2(I) K933 (62% Hyl in null vs 100% in WT). Significant decreases in hydroxylation of these residues are also seen in CyPB-deficient osteoblast collagen. It is generally accepted that hydroxylation of helical lysine residues is catalyzed by LH1. However, LH1 stability does not appear to be affected in *Ppib^−/−^* tissues based on western blot analysis (Figure 8D). Whether CyPB contributes to LH1 folding, or stabilizes its activity by binding, are among the possibilities to be investigated. Thus absence of CyPB from bone has critical direct and indirect effects on collagen, affecting both collagen folding and the activity of a major collagen-modifying enzyme, respectively. This gives CyPB a pivotal role in determining the structure of secreted collagen.

The site-specific changes in lysine hydroxylation seen in CyPB-deficient cells and tissues raises the possibility of an additional regulatory mechanism in bone, where there is apparent functional redundancy of LH activity directed at helical lysines not involved in crosslinking. Primary LH1 and LH2 deficiency demonstrated that LH1 hydroxylates lysines in the collagen triple helix while LH2 functions as the telopeptidyl lysyl hydroxylase [40]–[43]. However, collagen demonstrated variable decreases in Hyl content in bone from Ehlers-Danlos Type VIA patients (10–43% of normal) and a *Plod1* KO mouse (75% of wild-type), although specific helical lysines involved in crosslinks were always underhydroxylated based on decreased HP/LP crosslink ratios [44]–[46]. Thus, although LH1 appears to be required as the primary hydroxylase for helical domain crosslinking lysines in bone collagen, the relative roles of LH1, LH2 and LH3 at other helical sites and in other tissues is less well understood. LH3, which has LH, galactosyltransferase and glucosyltransferase activity *in vitro* and in culture [24], [27], [47], [48], could be the source of helical Hyl and even increased lysine glycosylation, in *Ppib* ^−/−^ bone [49]. Both a KO murine model for LH3 and a mouse with inactivated LH3 hydroxylation have faster gel migration of fibroblast type I collagen [50]. In the single human case of LH3 deficiency reported, the disaccharide derivative of pyridinoline crosslinks was absent in the patient's urine [28].

The intracellular hydroxylation of collagen helical K87, K930/933 and telopeptidyl lysine residues determines the collagen crosslink pathway. Crosslinks between collagen molecules in extracellular matrix are crucial to skeletal function because they contribute to matrix stability, bone strength and ductility. In both dominant OI caused by primary collagen defects and recessive OI caused by CRTAP and P3H1 deficiency, the overhydroxylation of collagen helical lysines leads to increased divalent DHLNL/HLNL and trivalent HP/LP collagen crosslink ratios [51]–[55]. In contrast, in *Ppib^−/−^* mice the underhydroxylation of K87 residues in tissue collagen results in substantial decreases in DHLNL/HLNL and HP/LP ratios in bone. Notably, these changes are similar to findings in primary LH1 deficiency, in which bone-derived urinary peptides reflect decreased hydroxylation of collagen lysine residues involved in crosslink formation [40], and decreased HP/LP ratios and increased total pyridinoline crosslinks were observed [40], [56], [57]. The cause of increased total crosslinks seen in *Ppib^−/−^* bone collagen is not clear. Possibly, decreased K87 hydroxylation and glycosylation may favor binding or activity of lysyl oxidase at this site [25], [58], [59].

Eyre and colleagues proposed that the collagen prolyl 3-hydroxylation modification supports matrix supramolecular assembly by fine-tuning the intermolecular alignment of collagen molecules to facilitate crosslink formation [55]. Our finding of severe reduction in collagen deposition into matrix is consistent with this hypothesis. However, we now understand that collagen secreted by CyPB-deficient osteoblasts has site-specific alterations in hydroxylation and glycosylation as well as absent P986 3-hydroxylation, which could also alter matrix assembly in addition to changes in collagen crosslinking and fibril structure. Furthermore, although this investigation focused on type I collagen, extracts from *Ppib* ^−/−^ bone show a substantial decrease in the quantity of type V collagen alpha chains (Figure 6F).

The effect of altered collagen crosslinking in *Ppib* ^−/−^ bone on mineralization remains to be explored. Bone from OI caused by collagen structural defects or CRTAP deficiency, whose crosslink pattern is opposite to this *Ppib* ^−/−^ mouse, is paradoxically hypermineralized. Collagen crosslinking and bone mineral crystallinity were strongly correlated in long bones of several congenic mouse strains [60]. On the other hand, increased HP/LP ratios correlate with the ultimate compressive strength of trabecular bone, but are independent of BMD [61], [62]. Comparison of mineralization in *Ppib* ^−/−^ and classical OI bone will provide insight into the interaction of bone crosslinks and mineralization, and could point to collagen modification as an intracellular pathway by which osteoblasts can actively influence bone mineralization.

Our initial goal in generating this mouse model of CyPB deficiency was to address the inconsistent findings of type I collagen lysyl and P986 hydroxylation among patients with type IX OI. Our analysis has revealed features of collagen biochemistry that have yet to be addressed in patients with type IX OI, in which all four sets of analyses were limited to collagen from fibroblast cultures. First, the extent and distribution of collagen helical modification in murine *Ppib* ^−/−^ samples differed between cells and tissues, with the overmodification of type I collagen from cultured cells due to increased glycosylation of a normal number of hydroxylysine resides. Future site-specific examination of tissue samples from type IX OI will be required to determine whether LH1 function is compromised and whether compensatory hydroxylation occurs. Second, type I collagen 3-hydroxylation differs between humans and mice with CyPB deficiency. In cultured cells of type IX OI patients, α1(I) P986 3-hydroxylation is normal in two moderately severe cases and reduced to about 30% in two lethal infants, while tissues and cells from both murine models have totally abolished P986 3-hydroxylation. Thus, our data suggests that the redundancy for CyPB's role in collagen folding may not apply to its role in 3-hydroxylation of the A1 site of type I collagen in mice. Tissue studies of type IX OI probands will be critical to determine whether the role of CyPB in 3-hydroxylation by the CRTAP/P3H1 complex is fully rescued by redundancy in human cells with total absence of CyPB, but only partially rescued in the presence of truncated CyPB.

## Materials and Methods

### Generation of *Ppib*-null mice

ES cell gene trap lines with a β-geo reporter construct inserted in intron 1 of the *Ppib* gene were produced by BayGenomics (UCSF, CA) and obtained from the Mutant Mouse Regional Resource Center (Davis, CA) [63]. The gene trap construct contains intron 1 and a portion of exon 2 to include splice acceptor sequence from the mouse *En2* gene, followed by a β-galactosidase/neomycin (β-geo) reporter-selection cassette and SV40 polyadenylation signal. Cells were expanded for isolation of mRNA and quantitation of *Ppib* expression. A heterozygous clone from cell line RST139 was shown to have half-normal *Ppib* expression by real-time RT-PCR, and was injected into C57BL/6 blastocysts. Founders were generated by mating chimeric males with 129/P2/OlaHsd females to retain the 129 background of the ES cell line. A second line was generated for experiments by backcrossing F1 mice into C57BL/6 for 5 generations.

Genomic DNA for genotyping was isolated using the Red Extract-n-Amp tissue PCR kit (Sigma) and amplified by hemi-nested PCR using a sense primer located in *Ppib* exon 1 (5′-TGCCCGGAC CCTCCGTGGCCAACGATAAGA-3′), an antisense primer corresponding to intron 1 of *En2* (5′-GGCATCTCCCCTTCAGTCTTCCTGTCCAGG-3′), and an antisense primer downstream of the inserted construct internal to *Ppib* intron 1 (5′-GGGGGGCTGGGGGAGTCTGGGTTATTCTCT-3′). Complete absence of *Ppib* transcripts was verified by real-time RT-PCR of mRNA isolated from femoral tissue and skin of F2 *Ppib* homozygous knock-out mice. Animal care and experiments were performed in accordance with a protocol approved by the NICHD ACUC committee.

### Skeletal staining, growth curves, X-ray, DXA analysis

For skeletal staining, skin and viscera were removed from dead P1 newborn pups. Pups were fixed in 95% ethanol for 7 days and stained with 0.3% Alcian Blue 8GS and 0.1% Alizarin Red S [64]. Excess stain was removed with 1% KOH and increasing concentrations of glycerol. For growth curves, mice were fed regular rodent chow and weighed weekly from 3 to 24 weeks of age. Skeletal characteristics of F6 wild-type, heterozygous and homozygous *Ppib*-null littermates were analyzed at age 8 weeks. Radiographs were performed by Faxitron (30 kV for 1 min). Areal bone mineral density (aBMD) scans of mouse femurs were acquired using a GE Lunar PIXImus2 (GE Healthcare) and internal calibration standards. To determine femoral length, femora were dissected and cleaned of soft tissue, leaving the epiphyses intact. Femurs were measured from the proximal head to the distal end of the medial and lateral condyles with a digital caliper.

### Micro-computed tomography, mechanical testing

Left femora of 8 week-old mice were analyzed by μQCT for both structural and mineral parameters using a SkyScan1174 compact micro-CT scanner (MicroPhotonics) operating at 50 kV with an X-ray source current of 800 µA, according to manufacturer directions. BMD was calibrated with hydroxyapatite phantoms. Morphometric analyses of trabecular and cortical regions was performed using CTAn software (v.1.13) and 3D images were generated using CTvol software (v.2.2). The trabecular region of interest (ROI) was located just proximal to the distal femoral growth plate and extended 10% total femoral length. The diaphyseal cortical ROI centered on the femur midpoint, spanning 15% total femoral length.

Femora were loaded to failure in four-point bending as previously described [65]. Femora were loaded to failure in four-point bending at 0.5 mm/s in the anterior-posterior direction with the posterior surface under tension using a servohydraulic testing device (MTS 858; MiniBionix; MTS Systems Corporation). Force was recorded by a 50lb load cell (Sensotec) and vertical displacement by an external linear variable differential transducer (Lucas Schavitts) at 2000 Hz. Load-displacement curves were used to calculate stiffness, yield load, yield displacement, ultimate load, failure displacement, post-yield displacement, and energy to failure.

### Cell culture

Primary fibroblast (FB) and calvarial osteoblast (OB) cultures were derived from 3-day old pups by standard procedures [66]. Cells from digestions 3–5 were plated at a density of 5,000 cells/cm^2^ and cultured in αMEM with 10% FBS, 2 mM glutamine, 1% pen-strep and 8% CO_2_. FB cultures were derived from dermal tissue dissected from the abdomens of newborn pups. FB were allowed to grow out from dermal samples for 2 weeks, released by trypsin digestion and cultured in DMEM containing 10% fetal bovine serum, 2 mM glutamine, 1% pen-strep and 5% CO_2_.

### Analysis of gene expression

Total RNA was extracted from cell cultures or tissues dissected from F6 mice using TriReagent (Molecular Research Center) according to the manufacturer's protocol. Total RNA was treated with DNA-free (Life Technologies), then reverse-transcribed using a High Capacity cDNA Archive Kit (Life Technologies). Real-time RT-PCR was performed using Taqman Assays on Demand (Life Technologies, *Ppib*, Mm00478295_m1; *Plod1*, Mm01255760_m1; *Plod3*, Mm00478798_m1; *Glt25d1*, Mm00600638_m1; *Glt25d2*, Mm01290012_m1; *Gapdh*, Mm99999915_g1). Relative expression of genes of interest was measured in triplicate, normalized to *Gapdh* transcripts, and quantified relative to wild-type calvarial OB.

### Western blot analysis

Two independent fibroblast (FB) and osteoblast (OB) cultures for each genotype were lysed in RIPA buffer (150 mM NaCl, 1% NP-40, 0.5% Na-deoxycholate, 0.1% SDS, 50 mM Tris, pH 7.4) supplemented with protease inhibitor cocktail (Sigma). Protein concentration was determined using the BCA Protein Assay Kit (Thermo Scientific). Samples (15 µg protein) were subjected to SDS-PAGE on 10% acrylamide gels under denaturing conditions and electroblotted onto nitrocellulose membranes. The membranes were blocked overnight in 5% non-fat milk in TBST. After washing in TBST, membranes were incubated overnight at 4°C in TBST containing 2.5% non-fat milk and primary antibody (diluted 1∶1000). After washing in 1× TBST, membranes were incubated with the corresponding IRDye infrared secondary antibody (diluted 1∶10,000) (LI-COR Biosciences). Proteins were visualized using an Odyssey Infrared Imaging System (LI-COR Biosciences). Quantitation of proteins was performed using the Odyssey 3.0.30 software with normalization to Actin levels. The analysis was repeated to ensure reproducibility. The primary antibodies used include anti-P3H1 (Abnova), rabbit anti-actin (Santa Cruz Biotechnology), rabbit anti-CyPB (Abnova), rabbit anti-PLOD1 (Santa Cruz Biotechnology), rabbit anti-PLOD3 (Proteintech), and goat anti-GLT25D1 (Santa Cruz Biotechnology). Anti-mouse CRTAP antibody was a generous gift from Dr Brendan Lee, Baylor College of Medicine.

### Biochemical analysis of type I collagen

Steady-state collagen analysis was performed as previously described [67]. Collagens were prepared by pepsin digestion (50 µg/ml) of procollagen samples, separated on 6% SDS-urea- polyacrylamide gels and visualized by autoradiography.

For analysis of collagen modification in cell culture, confluent FB and OB were stimulated for collagen synthesis in DMEM or αMEM containing 0.1% FBS and 100 µg/ml ascorbate for three days, with daily collection. Collected medium was buffered with 100 mM Tris-HCl, pH 7.4, and cooled to 4°C. Protease inhibitors were added to the following final concentrations: 25 mM EDTA, 0.02% NaN_3_, 1 mM phenylmethylsulfonylfluoride, 5 mM benzamidine, and 10 mM N-ethylmaleimide. Procollagens were precipitated from media with ammonium sulfate overnight at 4°C. Procollagen was collected by centrifugation, resuspended in 0.5 M acetic acid and digested with 0.1 mg/ml pepsin at 4°C overnight. Selective salt precipitation of collagen with 0.9 M NaCl in 0.5 M acetic acid was performed twice. Purified collagen samples were resuspended in 0.5 M acetic acid, dialyzed against 5 mM acetic acid overnight and lyophilized before further analyses.

Differential Scanning Calorimetry (DSC) scans were performed as previously described [68]. Thermograms were recorded in 0.2 M sodium phosphate, 0.5 M glycerol, pH 7.4, from 10 to 50°C at 0.125 and 1°C/min heating rates in a Nano III DSC instrument (Calorimetry Sciences Corporation).

Analysis of tissue-derived collagens was performed using skin, femora and humeri from 2 month old mice (n = 5). After marrow was flushed with cold PBS, bone samples were pulverized in liquid N_2_, demineralized with EDTA at 4°C for 2 weeks, and lyophilized. Skin dissected from the backs of mice was minced and lyophilized after removal of hair, fat and muscle. Two mg of the dried samples were then reduced with standardized NaB^3^H_4_, hydrolyzed with 6N HCl and subjected to amino acid and cross-link analyses as previously described [69]–[71]. The extent of Lys hydroxylation in collagen was calculated as hydroxylysine (Hyl)/hydroxyproline (Hyp)×300 (i.e. ∼300 residues of Hyp/collagen). The cross-link precursor aldehydes and reducible cross-links were measured as their reduced forms and all cross-links were quantified as moles/mole of collagen.

### Characterization of collagen post-translational modifications

Collagen 3-hydroxylation was analyzed by in-gel tryptic digestion of SDS-PAGE-purified type I collagen alpha chains. Electrospray mass spectrometry was performed on the tryptic peptides using an LCQ Deca XP ion-trap mass spectrometer equipped with in-line liquid chromatography (Thermo Finnigan) using a C8 capillary column (300 µm×150 mm; Grace Vydac 208MS5.315) eluted at 4.5 µl per min.

Site-specific modification of lysyl residues was determined by LC/MS/MS on a Waters Q-Tof Premier mass spectrometer coupled to a nanoACQUITY UPLC system (Waters Corporation) as reported [25]. Tryptic peptides containing Lys residues, their hydroxylated and/or glycosylated forms were identified from the LC/MS/MS analyses using manual interpretation of the MS/MS spectra. Relative quantitation of lysine (Lys), hydroxylysine (Hyl), galactosyl-Hyl (G-Hyl) and glucosylgalactosyl-Hyl (GG-Hyl) at a specific glycosylation site was performed by dividing the total ion abundance determined for each species by the sum of the ion abundances of all observed species containing that particular site.

### Collagen folding assays

An intracellular collagen folding assay was performed as described [17]. Confluent cells were stimulated overnight in media with 10% FBS and 100 µg/ml ascorbic acid, and then incubated in serum free media containing 100 µg/ml ascorbic acid for 2 hr. Cells were pulsed with 1.4 µCi/ml ^14^C-proline for 15 min to label procollagen chains, followed by collection of the cell layer every 5 min. Each sample was digested for 2 min at 20°C with 0.2% Triton X-100, 100 µg/ml trypsin, and 250 µg/ml chymotrypsin in PBS (Sigma). Digestions were stopped by addition of 1 mg/ml soybean trypsin inhibitor (Sigma). Samples were precipitated overnight, collected by centrifugation, electrophoresed on 3–8% Tris-acetate gels (Life Technologies) and quantitated by densitometry of autoradiograms. Assays were performed in duplicate on two independent cultures for each genotype.

### Collagen synthesis and secretion kinetics

For pulse-chase assays, performed as described [72], wild-type and homozygous *Ppib*-null FB were grown to confluence. For each cell line, two wells were used for cell counts. Procollagens were harvested at the indicated times, digested with pepsin and precipitated. Samples were loaded for equivalent cell number on 3–8% Tris-acetate gels. Collagen alpha chains were quantitated by densitometry and expressed as the percent secreted at each time point, as determined by (media)/(cell + media)×100.

### Collagen matrix deposition

Wild-type and homozygous *Ppib*-null FB and OB were grown to confluence and stimulated every other day for 14 days with fresh DMEM (fibroblasts) or αMEM (osteoblasts) containing 10% FBS and 100 µg/ml ascorbic acid, as described [73]. Matrix collagens were sequentially extracted at 4°C, with neutral salt for newly incorporated collagen, then acetic acid for collagens with acid-labile cross-links, and, finally, by pepsin digestion for collagens with mature cross-links [74]. All fractions were electrophoresed on 6% polyacrylamide-urea-SDS gels. Samples were loaded for equivalent densitometry signal; the total signal for each fraction was calculated by adjusting the gel signal by the total volume of that fraction. In separate experiments, quantitation of matrix deposited in culture was performed using Raman microspectroscopy. Cultures were fixed in 1% paraformaldehyde and analyzed as previously described [36]. Matrix collagen:cell organics ratios were evaluated from decomposition of corrected spectra of collagen-free cytoplasm and purified collagen in the amide III spectral region.

### TEM analysis of murine dermal collagen fibrils

A dermal biopsy was obtained from abdominal skin of wild-type and homozygous null mice, then processed as described [75]. Representative areas of the stained grids were photographed in a Zeiss EM10 CA transmission electron microscope (JFE Enterprises).

## Supporting Information

Figure S1 (A) Quantitation of *Ppib* expression in gene-trapped ES cells by real-time RT-PCR. Knockout founders were generated from cell line RST139. (B) Deposition of type I collagen by fibroblasts into extracellular matrix in culture. Post-confluent cultures were pulsed for 24 hr, followed by serial extraction of incorporated collagens from the media (M), neutral salt (NS), acid soluble (AA, immaturely crosslinked) and pepsin soluble (P, maturely crosslinked) fractions of the matrix. Samples were loaded for equivalent signal, and fractions were quantitated by densitometry of autoradiograms following PAGE analysis.
(TIF)

Table S1 Micro-CT analysis of mouse femora. Femora of 2-month male *Ppib^−/−^* mice display altered structural parameters of trabecular and cortical bone compared to wild-type mice.
(DOC)
