# Supplementary figures and images for: Abnormal Type I Collagen Post-translational Modification and Crosslinking in a Cyclophilin B KO Mouse Model of Recessive Osteogenesis Imperfecta
Source: PLoS Genet. 2014 Jun 26;10(6):e1004465. doi: 10.1371/journal.pgen.1004465 (PMC4072593; doi:10.1371/journal.pgen.1004465)

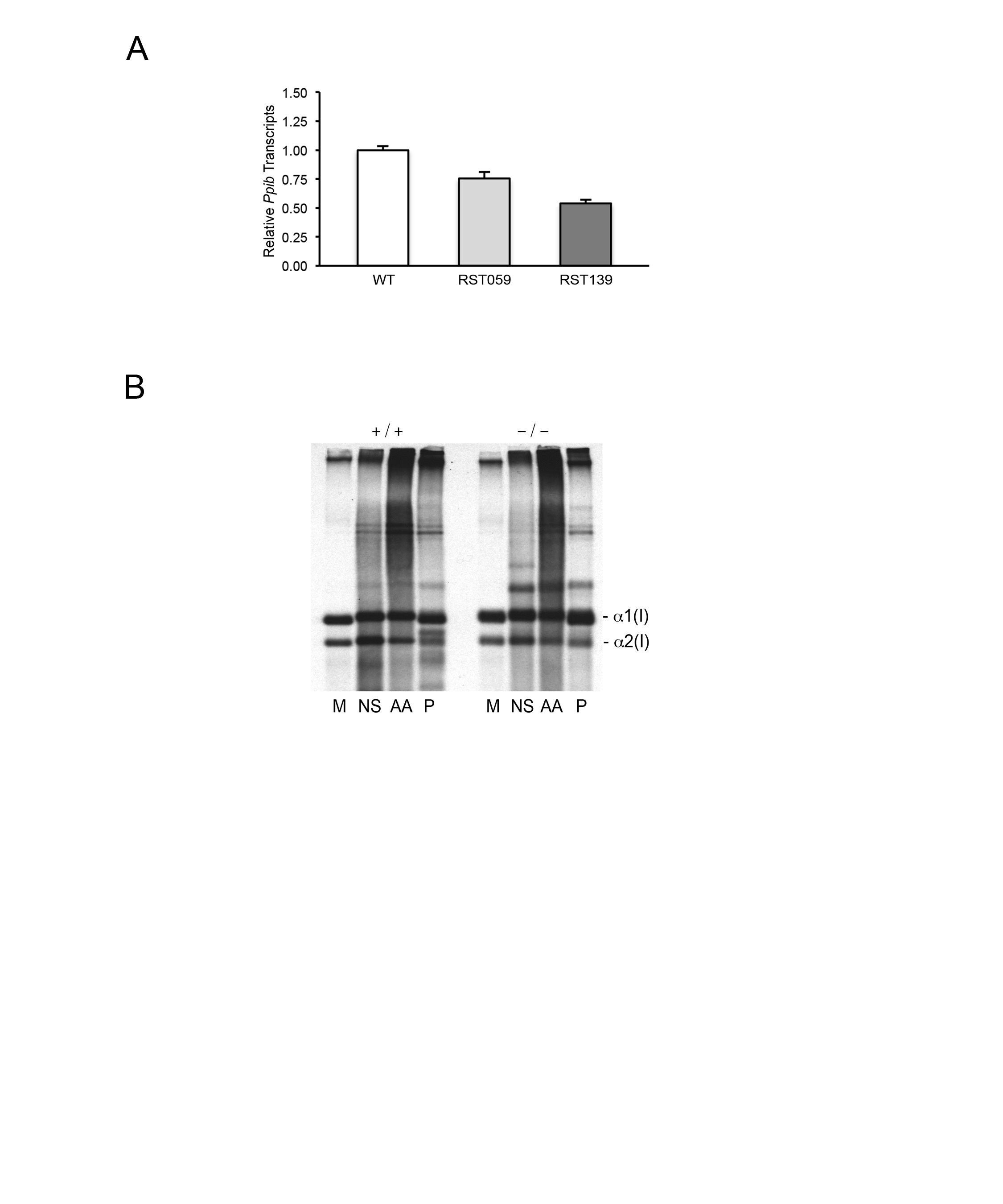

Supplement: Figure S1 — (A) Quantitation of Ppib expression in gene-trapped ES cells by real-time RT-PCR. Knockout founders were generated from cell line RST139. (B) Deposition of type I collagen by fibroblasts into extracellular matrix in culture. Post-confluent cultures were pulsed for 24 hr, followed by serial extraction of incorporated collagens from the media (M), neutral salt (NS), acid soluble (AA, immaturely crosslinked) and pepsin soluble (P, maturely crosslinked) fractions of the matrix. Samples were loaded for equivalent signal, and fractions were quantitated by densitometry of autoradiograms following PAGE analysis. (TIF) [file pgen.1004465.s001.tif]
