# Supplementary material for: Abnormal Type I Collagen Post-translational Modification and Crosslinking in a Cyclophilin B KO Mouse Model of Recessive Osteogenesis Imperfecta
Source: PLoS Genet. 2014 Jun 26;10(6):e1004465. doi: 10.1371/journal.pgen.1004465 (PMC4072593; doi:10.1371/journal.pgen.1004465)
Supplement: Table S1 — Micro-CT analysis of mouse femora. Femora of 2-month male Ppib−/− mice display altered structural parameters of trabecular and cortical bone compared to wild-type mice. (DOC) [file pgen.1004465.s002.doc]

**Table S1** Micro-CT analysis of Mouse femora

|  |  | **+/+** | **+/-** | **-/-** |
| --- | --- | --- | --- | --- |
| **TRABECULAR PARAMETERS** |  |  |  |  |
| Percent Bone Volume (%) | BV/TV | 15.963 ± 7.473 | 16.988 ± 6.965 | 8.131 ± 4.891 *† |
| Bone Surface/volume ratio (1/mm) | BS/BV | 95.931 ± 24.789 | 95.572 ± 22.484 | 122.706 ± 28.150 *† |
| Bone Surface density (1/mm) | BS/TV | 13.808 ± 3.403 | 14.975 ± 3.694 | 8.843 ± 3.730 *† |
| Trabecular Thickness (mm) | Tb.Th | 0.056 ± 0.014 | 0.056 ± 0.011 | 0.046 ± 0.008 † |
| Trabecular Number (1/mm) | Tb.N | 2.721 ± 0.720 | 2.935 ± 0.839 | 1.694 ± 0.846 *† |
| Trabecular Separation (mm) | Tb.Sp | 0.186 ± 0.035 | 0.179 ± 0.033 | 0.258 ± 0.077 *† |
| Bone Mineral Density (g/cm^3^) | BMD | 0.224 ± 0.043 | 0.228 ± 0.036 | 0.176 ± 0.026 *† |
|  |  |  |  |  |
| **CORTICAL PARAMETERS** |  |  |  |  |
| Cortical area (mm^2^) | Ct.Ar | 0.613 ± 0.072 | 0.620 ± 0.071 | 0.530 ± 0.068 *† |
| Marrow area (mm^2^) | M.Ar | 0.966 ± 0.076 | 0.946 ± 0.069 | 1.058 ± 0.163 † |
| Total tissue area (mm^2^) | T.Ar | 1.579 ± 0.117 | 1.562 ± 0.079 | 1.584 ± 0.192 |
| Cortical thickness (mm) | Ct.Th | 0.155 ± 0.015 | 0.156 ± 0.018 | 0.131 ± 0.016 *† |
| Bending moment of Inertia (mm^4^) | MOI | 0.092 ± 0.017 | 0.092 ± 0.014 | 0.082 ± 0.015 |
| Bone Mineral Density (g/cm^3^) | BMD | 1.060 ± 0.059 | 1.063 ± 0.053 | 1.022 ± 0.068 |

Average ± Standard Deviation; * p < 0.05 -/- vs. +/+; † p < 0.05 -/- vs. +/-
